# Potential Application of Ionic Liquids for Electrodeposition of the Material Targets for Production of Diagnostic Radioisotopes

**DOI:** 10.3390/ma13225069

**Published:** 2020-11-10

**Authors:** Maciej Chotkowski, Damian Połomski, Kenneth Czerwinski

**Affiliations:** 1 Faculty of Chemistry, University of Warsaw, Pasteura 1, 02-093 Warsaw, Poland; dpolomski@chem.uw.edu.pl; 2 Biological and Chemical Research Centre, University of Warsaw, Żwirki i Wigury 101, 02-089 Warsaw, Poland; 3 Radiochemistry Group, University of Nevada, Las Vegas, NV 89154, USA

**Keywords:** RTIL, ionic liquid, radioactive elements, electrochemistry

## Abstract

An overview of the reported electrochemistry studies on the chemistry of the element for targets for isotope production in ionic liquids (ILs) is provided. The majority of investigations have been dedicated to two aspects of the reactive element chemistry. The first part of this review presents description of the cyclotron targets properties, especially physicochemical characterization of irradiated elements. The second part is devoted to description of the electrodeposition procedures leading to obtain elements or their alloys coatings (e.g., nickel, uranium) as the targets for cyclotron and reactor generation of the radioisotopes. This review provides an evaluation of the role ILs can have in the production of isotopes.

## 1. Introduction

Modern medicine gives an opportunity to detect lesions at an early stage of development. It is possible thanks to the access to modern diagnostic methods, including radio imaging. The number of the medical examinations with application of radionuclides is large and growing. For instance, for the US, in 1973, 3.5 million nuclear medicine procedures were performed, increasing to 17 million in 2005 [1]. At the same time, thanks to new short-lived PET (positron emission tomography) diagnostic radiopharmaceuticals (e.g., ^124^I, ^89^Zr) there was a reduction on the negative impact to the surrounding tissues. Application of short lived isotopes for imagining (T_1/2_~min–h) has advantage over longer lived isotopes (T_1/2_~days) due to lower effective ingestion dose factor. For instance, for technetium, ^99m^Tc (T_1/2_ = 6 h, E_γ_ = 140 keV) has 1.68 × 10^−11^ Sv∙Bq^−1^ and is much lower than for its chemical analogue ^186^Re (T_1/2_ = 3.72 day, E_γ_ = 137 keV (9.4%), E_β(max)_ = 1069 keV (70%)); 1.55 × 10^−9^ Sv∙Bq^−1^ [2].

In nuclear medicine, the most popular isotopes are ^99m^Tc and ^131^I. About 80% of all radio-diagnostic procedures involves ^99m^Tc [3]. These and other isotopes are listed in Table 1. Nowadays, the aforementioned ^99m^Tc and ^131^I are mainly produced in nuclear reactors, others like ^44^Sc or ^55^Co in cyclotrons. Increasing interest in their use affects the need for their effective production. The latter factor is directly connected with the high quality targets suitable for irradiation. Although for some elements their deposition procedures understood, but this is not true, especially for new PET radioisotopes. For these new imaging agents electroplating protocols for targetry development can aid in their production and separation.

Only a few element targets for nuclear medicine naturally occur as single stable isotopes. Unfortunately, most elements are the mixtures of the isotopes. Cyclotron production protocols often require usage of enrichment targets materials which are expensive and difficult to obtain. For instance 50 mg of ^100^Mo (abundance: 14.77%) powder costs approximately 1000 € while 50 g of natural Mo is 50 €. This colossal price difference between natural and enrichment target isotopes justifies the search for efficient methods of target materials deposition and their recovery. The available activity of produced isotopes from cyclotron depends on their half-life and cross sections of the intended target atoms. For the most popular nuclear reaction the latter factor is relatively low, at level of mbarns [7,8,9,10]. In turn, this parameter for ^235^U(n, nx)SF reaction is much higher (c.a. 581 b for thermal neutrons) but this reaction requires strong neutron sources available in nuclear reactors. For both cases, cyclotron and nuclear reactor, significant increase of the temperature of target material is observed. For instance, in experimental reactor Maria in Poland high enrichment uranium (over 90% UAl_~3_ target) is irradiated for Mo-99 production with average fission power at level of 165–190 kW. The activity of this isotope at the end of irradiation is 7.5–8 kCi [11,12,13]. LEU-based targets (U, UAl_x_, U_x_Si_y_, U-alloy and U_3_O_8_) are also under development for radiopharmaceutical manufacturing [14].

The atom density of the metallic target provide higher atom densities in comparison to metal oxide target as can be seen in Table 2. Except calcium and strontium, for all mentioned couples the atomic ratio of the number of atoms in metal to metal oxide is higher. It is especially visible for high density materials, e.g., molybdenum or uranium. It means that for the same volume target and the same proton or neutron flux in cyclotrons or nuclear reactors, there is generally a more efficient creation in radionuclides metal targets than oxides. Sometimes deposition of the elements in their elemental forms admittedly seems to be attractive but not all of them are suitable for this process. During irradiation of the target disc may heat up to the temperatures even above 200 °C [15] therefore the materials characterized by low melting points, e.g., selenium, tellurium, pose major technological challenges.

An advantage of a metallic or intermetallic alloys over their oxides includes superior improved matrix compatibility and thermal conductivity. For instance the latter parameter for metallic uranium equals c.a. 26 Wm^−1^ K^−1^ at 300 K [16], approximately three times higher than for uranium dioxide [17]. Sometimes, especially for metalloids as tellurium, an aluminum additive is used to increase the heat transfer characteristics of targets [7]. Not all elements can be considered as suitable heat transfer additive candidates. The nuclear properties of the elements need to be considered. Elements located in d-block have very good thermal properties but can be activated in a cyclotron proton flux. For the example; ^nat.^Pt→^194^Au (T_1/2_ = 38 h) and ^nat.^Pd→^104^Ag (T_1/2_ = 69 min). The elements Pt and Pd would have suitable thermal transfer properties but may significantly increase the activity of irradiated sample.

For some from aforementioned elements (e.g., CaO, SrO), their oxides are used as the targets suitable for irradiation. Biswal et al. [18] discussed the role of surfactants in electrochemical deposition of Co−Ni−Cu systems. These additives (cetyltrimethylammonium bromide and dodecyltrimethylammonium bromide) play significant role in determination of the surface morphology, crystal structure, and the electrochemical properties of electrodeposited materials. Therefore consideration needs to be given to the resulting morphology associated with deposition and target preparation.

Thickness of the target materials electrodeposited onto backing plate are at level of microns, up to 100 μm. This layer should be thick enough to minimalize copious production of unwanted radionuclides in the backing plate materials. For copper targets the isotope ^65^Zn, a high gamma emitter with relatively long half-life of 245 days, is produced [7]. Very often aluminum backing is preferred because of its simplicity of handling, adequate thermal properties and acceptable nuclear interactions.

In 1977 Van Den Bosch et al. [7,19] described the requirements for ^124^Te target material for ^124^I cyclotron production. They are summarized below:

1. Radiation and thermal stability of the target and target support under irradiation;
2. Adequate heat dissipation and thermal conductivity of these materials;
3. Simple and almost complete separation of produced radionuclide from target material within a short period of time, preferably less than half an hour;
4. Simple and high efficient (99% per irradiation and separation) reprocessing of the target;
5. The chemical state of the produced radionuclide should not handicap any in vivo application or labelling procedure.

It should be also added:

6. Smooth, dendrite free surface with appropriate granulometry.

These rules can be generally adopted for very expensive enriched target materials on most cyclotron procedures. Of course, the form will vary depending on the physical and chemical characteristics of the target material.

Elements with relatively high values of *E*° [20,21] can be deposited directly. Others like calcium or strontium cannot be directly deposited from aqueous solutions onto solid electrodes. Electrodeposition of elemental layers of nickel is achievable; calcium, scandium, yttrium, strontium, or uranium do not have suitable electrochemical windows to form thick enough metals films in aqueous solutions.

In the literature one may find the procedures leading to electrodeposit trace amounts of uranium as a sources for alpha-particle spectrometry. For instance, Oh et al. [22] examined ammonium chloride, ammonium oxalate and ammonium sulphate as the electrolyte solutions for U, Pu and Am deposition. Krmpotića et al. [23] described the procedures leading to obtain alpha sources from acidified (pH~2) aqueous solutions of sulfates or oxalates. For uranium these conditions can be suitable for oxide layer formation, however the applications to alpha spectroscopy preclude formation of thick targets. For metallic targets of uranium, other systems are needed.

For chemically active elements non-aqueous solutions are proposed. For instance Kumbhar and Lokhande electrodeposited yttrium from the mixtures of ethanol and various acids or salts [24]. Yttrium, barium, and copper coatings were investigated Ondono-Castillo et al. [25]. These authors obtained YBa_2_Cu_3_O_7_-_δ_ layers in dimethyl sulfoxide. The latter solution was used by Shirasaki et al. [26] for uranium electrodeposition. These authors obtained U layers onto Au electrode at −2.2 V versus ferrocene/ferrocenium reference electrode.

Other elements can be electrodeposited from non-aqueous solutions. In the 1930s Audrieth and Nelson [27] discussed in their review article the electrochemical methods for metals (Zn, Cd, Pb, Sn, Ni, Co) deposition from various organic solvents. Contemporary publication of Panzeri et al. [28] has shown the possibility of the electrodeposition of zinc from a chloride free based on ethylene glycol and acetate salts. Zinc layers were obtained on the steel working electrode at current densities of −2 mAcm^−2^ to −16 mAcm^−2^. In turn, Ta et al. [29] discussed the mechanism of Zn (and Mg) electrodeposition from acetonitrile-based solutions.

Molten salt are suitable solution environments for elemental electroplating [30,31,32,33,34] but their disadvantage is the requirement of high temperature at which the systems are maintained (e.g., LiCI-KCl at 450 °C [30], NaCl-KCI at 700 °C–900 °C [31], NaF-AlF_3_ or KF-AlF_3_ at 750 °C–980 °C [32], LiCI-KCl at 400 °C–550 °C [34]). A promising alternative to molten salts are ionic liquids with their programmable properties [35]. To achieve good adhesion of deposited layer of the element or its alloy to the substrate, the electrodeposition process is often at elevated temperatures, mainly up to 120 °C. For isotope target production, selected electrodeposition protocols will be discussed in later part of the article.

Thermal stability of ILs has been tested by various authors. For example, Kosmulski et al. [36] investigated imidazolium-based phosphates, hexafluorophosphates or triflates. A significant increase of their decomposition rate is observed at or above 200 °C. In turn, Malton et al. [37] in their review article discussed short term thermal stability of various types of ammonium- and imidazolium-based ionic liquids. All of them have been shown to be stable at temperatures above 120 °C and most of them above 250 °C.

Other type of ionic liquids was of interest of Delgado-Mellado et al. [38]. They investigated thermal stability of choline based deep eutectic solvents (DES). Mass loss of one of the most popular DES, ChCl + Urea (1:2), after 20h heating at 70 °C was low and equaled 1.52wt%. In general, based on theromgravimetric analyses they ranked DES solvents from the least to the most stable:

ChCl:EG < ChCl:Mal < ChCl:Lev < ChCl:Phenylac < ChCl: Phenylprop < ChCl:Glyce < ChCl:Urea < ChCl:Gluc (all abbreviations of the ionic liquids are listed below the text).

Ionic liquids are attractive solvents for industrial usages in extraction and separation processes [39,40,41], gas adsorption [42,43,44,45], synthesis of metal nanoparticles [46,47,48], energy conversion and storage [49,50,51], and a biotransformation [52,53] or metal electrodeposition [54]. An interesting property of some ionic liquids is their ability to dissolve metal oxides (MO), Table 3. High solubility in ionic liquids based on choline cations is observed for PbO_2_ and ZnO. This feature of ILs is desirable to offset potential MO generation during electroplating. Depending on the structure of ILs, the mechanism of this reactions differs. For the ILs with labile hydrogen, e.g., [BMIm][HSO_4_], this process can be described as, Equation (1):

(1) $$\mathrm{MO}+\;2{\mathrm{HSO}}_{4}^{-}\;\to {\;M}^{2+}+\;2{\mathrm{SO}}_{4}^{-}+H_{2}O$$

For deep eutectic solvents based on ChCl a complexation reaction between metal and chloride in the presence of organic acids leads to chloro- or chlorooxometalates, MCl_x_^z−^, MOCl_y_^z−^. For ZnO, the reaction in the presence of urea and choline chloride can be described according to Equation (2) [55]:

(2) $$\mathrm{ZnO}+\;\mathrm{ChCl}+\mathrm{urea}\;\to \;\mathrm{ZnClO}{\left(\mathrm{urea}\right)}^{-}+{\;\mathrm{Ch}}^{+}$$

As reported Abbot et al. [56] the ability of DES based on choline chloride to dissolve metal oxides changes in the order malonic acid > urea > ethylene glycol.

Based on behavior described above, the application of ionic liquids for the formation of isotope targets is provided below. The conditions and chemical trends for electroplating elemental targets are identified. These conditions can be used to expand the potential pool of isotopes for medical applications and provide routes for preparation of targets for coupled separations and potential reuse.

## 2. Electrochemistry of Targets Elements

### 2.1. Calcium (Ca) & Strontium (Sr)

The most common calcium or strontium targets are made of their carbonates or oxides [7]. Both materials have their advantages and disadvantages. For instance calcium/strontium carbonates have worse thermal properties than the oxides but the latter are sensitive to carbon dioxide. Strontium targets can be prepared by sedimentation [57] although the most popular methods assumes powder pressing into a pellet (e.g., 4.14 MPa for 30 s). Due to extremely low standard redox potentials for Ca^2+^/Ca or Sr^2+^/Sr couples, E^⦵^ = −2.76 V or −2.89 V, respectively, electrodeposition of a metallic layer on solid materials from aqueous solutions is impossible. Both metals can be eventually electrodeposited from their fused salts at high temperatures.

Calcium electrochemistry in non-aqueous solutions is considered mainly in context of Ca-ion batteries [58,59]. To the best of our knowledge, only one article is directly devoted to electrodeposition of calcium in ILs. Biria et al. [60] characterized Ca films onto a copper electrode obtained from [EMIm][TfO]. These deposits were generated during many hours of consecutive cycles of plating and stripping at a current density of 0.55 mA∙cm^−2^. Plating potential was −0.15 vs. Ca│Ca^2+^ electrode. Noteworthy is the fact that these authors obtained smooth layer of 20 µm thick, which is very good from the point of view of potential application as a cyclotron target. For magnesium its salt has been characterized in non-aqueous solutions by Minakshi et al. [61].

Only a few articles deal with the electrodeposition of strontium in ionic liquids. For instance, Chen and Hussey [62], and Chen [63] reported electroreduction of Sr^2+^ complexed by dicyclohexano-18-crown-6 in ammonium, imidazolium or pyrrolidinium based bis((trifluoromethyl) sulfonyl)imides. The author stated that the best results were obtained for [N3331][Tf_2_N] due to its high electrochemical stability. At potentials below 2.9 V vs. Fc^+^│Fc, a well-defined reduction wave was observed. The working electrode was Hg film coated upon Pt. Later, Hori et al. [64] reported electrodeposition of strontium hydroxyapatite nanorods, SrHAp, on titanium/silicon in [TMA]Cl as the supporting electrolyte. The applied potential to Ti/Si electrode was −1.2 V vs. Ag│AgCl reference electrode. The authors stated that obtained results are similar to those observed in 0.1M KCl [64].

A characteristic feature of all strontium based deposits was their nanorod-like grains. These properties are preferable for biocompatible Sr-(hydroxy)apatite materials for bone filling but from the point of view of their potential application as a cyclotron targets such structures are not preferable. However these material, with their relatively high surface area, may act as porous targets for the selective extraction of produced isotopes [65].

### 2.2. Scandium (Sc) & Yttrium (Y)

The isotope ^45^Ti as a non-conventional radionuclide for PET imagining can be obtained by proton irradiation of a scandium target [4]. Scandium as well as Y are monoisotopic elements, hence no enrichment process is required and it dramatically decreases the costs of the target. Due to low standard redox potentials of Sc/Sc^3+^ and Y/Y^3+^ couples (E^⦵^ = −2.03 V, E^⦵^ = −2.31 V), these elements are not obtained in their elemental forms from aqueous solutions. Scandium as well as yttrium can be electrodeposited from non-aqueous systems [24,34]. From aqueous solutions both elements can be deposited as oxides or hydroxides [66]. Sc-Al alloys obtained in molten salts are known, but their adhesion to a substrate is unsatisfactory [34,66].

Only a few papers are devoted to electrodeposition of yttrium from ILs. Glukhov et al. [67] electrodeposited this element from ILs based on quaternary ammonium cations and triflate salts at 100 °C. No information of the morphology of obtained deposits is available.

Due to similar properties of Sc and Y to lanthanides, one may expect that their electrochemical behavior of these elements to be similar. Matsumiya [68] discussed application of ILs for electrodeposition rare elements, Nd and Dy. Granular and non-uniform Nd or Dy layers were obtained in [P2225][Tf_2_N], solutions at −3.4 V or −3.8 V respectively. Recent publication of Sanchez-Cupido et al. [69] delivers the information on the electrodeposition of Nd from [P66614][Tf_2_N], +0.1 mol∙kg^−1^ Nd([Tf_2_N])_3_ with small fraction of water (0.1% or 0.4 wt%). Obtained at −5.2 V vs. Fc^+^│Fc^0^, the deposit consisted of metallic Nd, Nd_2_O_3_ and NdF_3_. The authors stated that water content turned out to have significant impact on morphology of obtained deposits. Its decrease resulted in more smooth Nd layers with fewer cracks.

### 2.3. Nickel (Ni)

As a target material for cyclotron production of Co or Cu radioisotopes mainly enriched nickel targets with thickness of several dozen microns are used [7]. These materials are electrodeposited from various types of solutions.

**Figure 1:**
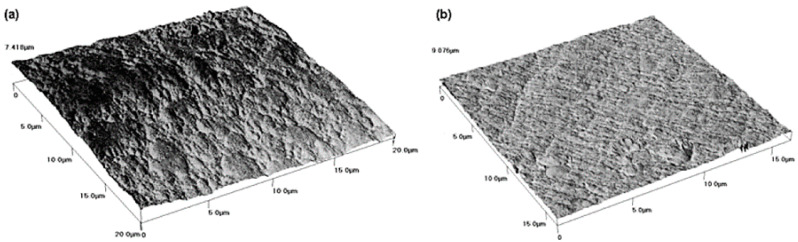
AFM images of Ni electrodeposited on Cu substrate from IL–Ni containing 0.5 M NiCl_2_ for various operating conditions: (**a**) 1 A∙dm^−2^, 50 °C, 30 min and (**b**) 0.65 A∙dm^−2^, 30 °C, 30 min, against a Ni anode. [Reproduction with permission from Florea, A.; Anicai, L.; Costovici, S.; Golgovicic, F.; Visanc, T., Surf. Interface Anal.; published by Wiley Interscience, 2010].

The literature devoted to electrochemical characterization of nickel compounds in non-aqueous solutions is rich [70,71,72,73,74,75]. Choline chlorine-based ionic liquids are extensively examined in context of their application as a medium for nickel electroplating. In 2010 Florea et al. [71] provided the information on the electrodeposition of Ni from 1ChCl:2Urea with: 0.42–1.68 M NiCl_2_∙6H_2_O or 0.71 M NiSO_4_. This element was deposited onto copper substrate in a two-electrode cell. Two anode materials were examined: nickel and graphite. The authors stated that application the first one is more favorable due to better control of Ni^2+^ concentration in the electroplating bath. From the point of view of potential application in cyclotron target production Ni as a counter electrode is not preferable due to its possible dissolution and subsequent electrodeposited. The applied current densities were in the range from 0.65 A∙dm^−2^ up to 15 A∙dm^−2^. Moreover Florea and co-workers determined deposition rate of the nickel layer at level of 0.1 to 0.4 µm∙min^−1^ for 4 to 15 A∙dm^−2^. Obtained deposits adhered to Cu, as can be seen in Figure 1.

Urea as a source of ammonia has been used in the precipitation of nickel hydroxide, Ni(OH)_2,_ Equation (3) [76].

(3) $${\mathrm{NH}}_{2}-\mathrm{CO}-{\mathrm{NH}}_{2}+{\;\mathrm{NiSO}}_{4}+\;3H_{2}O\;\to \;\mathrm{Ni}{\left(\mathrm{OH}\right)}_{2}↓\;+\;{\left({\mathrm{NH}}_{4}\right)}_{2}\cdot {\mathrm{SO}}_{4}+{\mathrm{CO}}_{2}$$

Ash B. et al. reported that pure α-Ni(OH)_2_ has been synthesized in various concentration of urea and at elevated temperatures [76].

**Figure 2:**
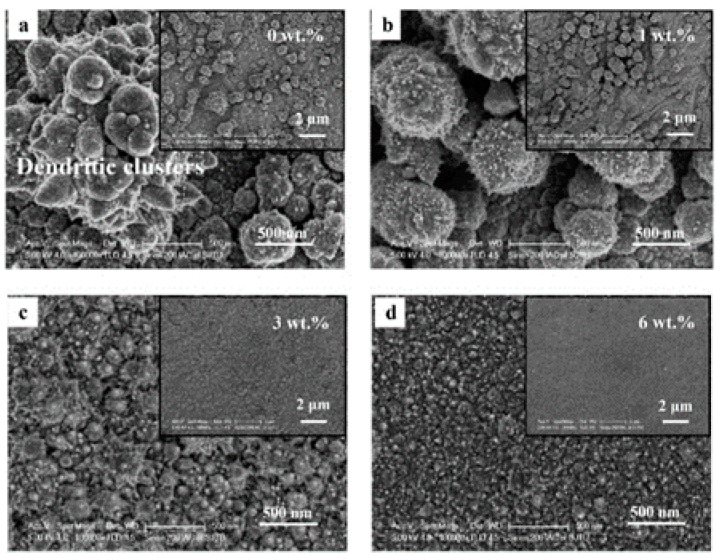
Micrographs of the Ni coatings electroplated from the ChCl-2Urea-(0.2 M)NiCl_2_ electrolyte containing (**a**) 0 wt.%, (**b**) 1 wt.%, (**c**) 3 wt.% and (**d**) 6 wt.% water at 318 K. [Reproduction with permission from Du, C.; Zhao, B.; Chen, X.-B.; Birbilis, N.; Yang, H., Sci. Rep.; published by Nature, 2016].

A significant impact of water content on the morphology of Ni layers was reported by Du et al. [77]. Figure 2 presents the micrographs of Ni deposits obtained from the solutions with various concentration of water. An increases of water in the electroplating bath resulted in a decrease in viscosity of the solution. This translates into increased current efficiency. As reported the authors, the deposits became denser and consisted of smaller Ni clusters.

Later, the same choline-based mixture was applied by Cherigui et al. [72] for electrodeposition of Ni nanostructures onto a glassy carbon electrode. They reported that a residual water content leads to Ni and Ni(OH)_2(ads)_. Lukaczynska et al. [78] reported significant influence of water content on electrodeposition of Ni from ChCl:urea solutions. Higher than 4.5%wt water content enhances decomposition of the solvents at potentials lower than −0.9 V vs. Ag as quasi-reference electrode (QRE). In turn, an influence of ethylenediamine as additive to ChCl:urea with NiCl_2_ electroplating solutions examinated Bernasconi and Magagnin [79]. They observed non-porous Ni layers onto Al substrate for Ni^2+^:ED molar ratio equaled 1:2 and current density of 10 mA∙cm^−2^.

Other DES-type ILs were examined Gong et al. [80] and Sun et al. [74]. Gong et al. electrodeposited Ni layers from water-stable (14.3%:85.7%mole fraction) betaine∙HCl:EG mixtures. The obtained deposit was dense and consisted small grains. In turn, Sun et al. [74] obtained Ni layers from choline chloride and ethylene glycol with NiCl_2_·6H_2_O and NaH_2_PO_2_ (0–50 mM) mixtures. They generated thin layers of deposits consisting of Ni and NiP_x_ nanoparticles suitable to catalysts for enhance hydrogen evolution region.

Zhu et al. [70] electrodeposited Ni films onto Cu substrate from ([BMPyr][Tf_2_N]) containing Ni([Tf_2_N])_2_ solutions. At current density of −0.046 mA∙cm^−2^ and 50 °C relatively smooth (although with a cracks) deposits were obtained. The authors observed that the grains size becomes bigger with increase of the temperature. Later, Zheng et al. [75] electrodeposited Ni layers from [BMIm][DBP] IL with NiCl_2_.

**Figure 3:**
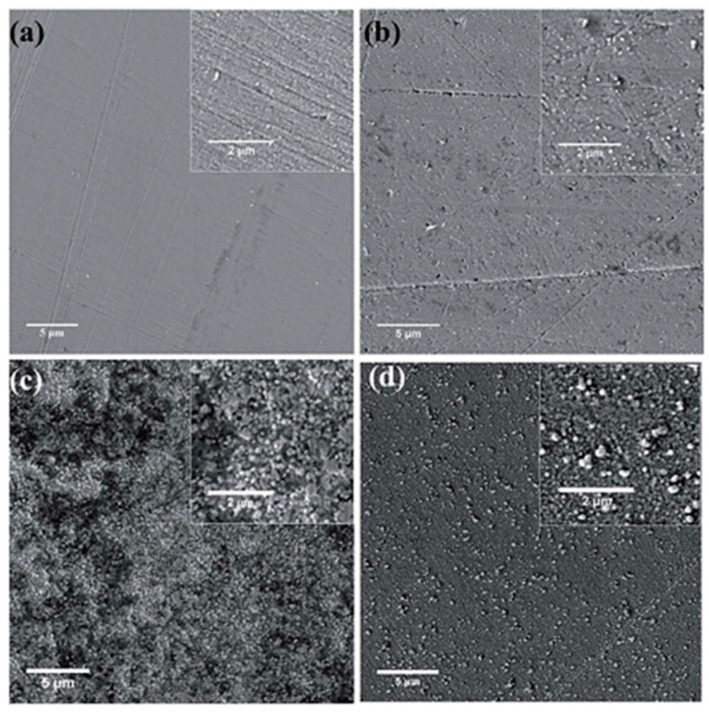
SEM images of nickel coatings formed on copper substrate at 363 K under potentiostatic electrodeposition at various potential (vs. Ag) (**a**) −0.40, (**b**) −0.50, (**c**) −0.60, (**d**) −0.70. [Reproduction with permission from Zheng, Y.; Zhou, X.; Luo, Y.; Yu, P., RSC Adv.; published by The Royal Society of Chemistry, 2020].

The coatings were obtained at various potentials (Figure 3). An analyses of the SEM imagines of Ni layers revealed that the best adherent film was observed at −0.4 V vs. Ag as a QRE. More negative potentials lead to less smooth coatings with cracks and additional spherical grains on the surface.

Nickel films can be successfully obtain from ILs. Many articles are focus on characterization of Ni deposits obtained especially from deep eutectic solvents. Electroplated layers are smooth, without visible cracks and adherent to the substrate.

### 2.4. Zinc (Zn)

Enriched zinc, ^68^Zn, is used for cyclotron production of ^68^Ga radioisotope [81]. Both pressed elemental zinc powder and electroplated metallic zinc have found application as a target materials. Electroplating of this element onto e.g., copper, silver or platinum supports can be made from various types of solutions, e.g., HCl/hydrazine or KOH/cyanide, with preparation yield at level of 90% [7,81].

**Figure 4:**
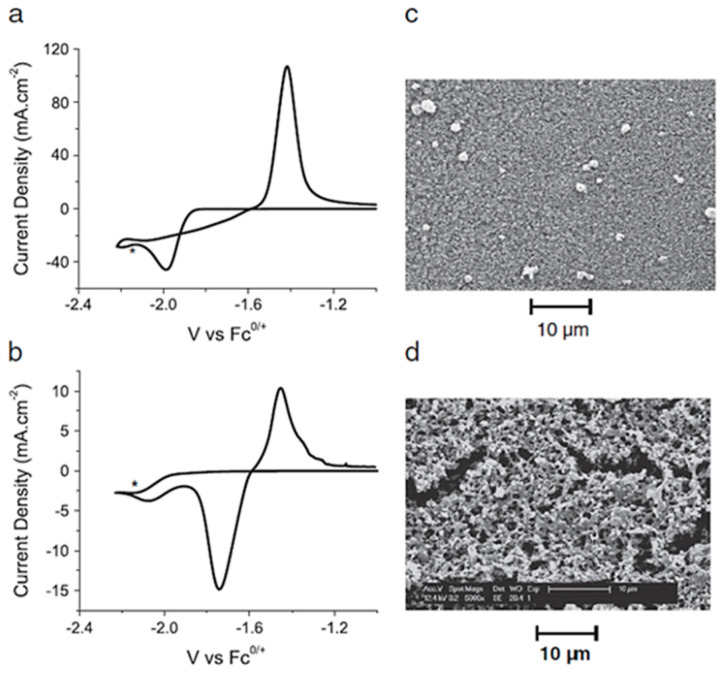
CVs and SEMs of 10 mol% Zn(dca)_2_ in [EMIm][DCA] on a GC working electrode held at −2.15 V vs. Fc^0/+^ (ferrocene/ferrocenium) (marked by *) for 10 min at different water contents: (**a**) CV of 3 wt% H_2_O, scan rate 100 mV∙s^−1^, (**b**) CV of 0.05 wt% H_2_O, scan rate 100 mV∙s^−1^, (**c**) SEM of 3 wt% H_2_O, (**d**) SEM of 0.05 wt% H_2_O. [Reproduction with permission from Simons, T.J.; Torriero, A.A.J.; Howlett, P.C.; MacFarlane, D.R.; Forsyth, M., Electrochem. Commun.; published by Elsevier, 2012].

Electrodeposition of metallic zinc from various types of ionic liquids and their mixtures is very well elaborated [82] and ref. therein. Primarily imidazolium based ILs have been intensively explored [82,83,84,85,86,87]. Simons et al. [84] investigated the influence of water content on the morphology of electrodeposited zinc coatings. Figure 4 presents cyclic voltammograms and SEM imagines of zinc dicyanide (Zn(dca)_2_) in EMIm][DCA] ionic liquid. Metallic layers were obtained at −2.15 V vs. Fc^+^│Fc. These authors determined that low water content (0.05%wt H_2_O) in IL results in poor adhesion of electrodeposited zinc film in opposite to the mixtures with 3%wt H_2_O for which closely packed needles is observed.

**Figure 5:**
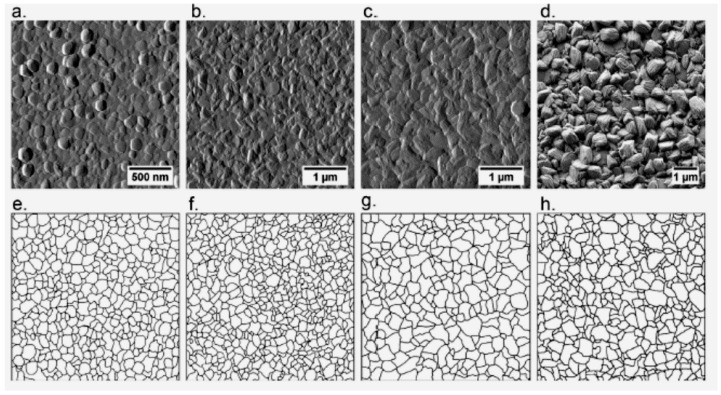
Representative in situ AFM and ex situ SEM images where (**a**–**c**) represents an AFM image (deflection) sequence of the zinc surface during electrodeposition within Zn(TfO)_2_/[BMIm][TfO] at a deposition overpotential of 445 mV vs. ECO after 400 Zn ML (418.4 mC∙cm^−2^), 1200 Zn ML (1255.2 mC∙cm^−2^), and 2800 Zn ML (2928.8 mC∙cm^−2^) of chargé passed, and (**d**) is a representative SEM image from the Zn surface after electrodeposition within Zn(TfO)_2_/[BMIm][TfO] within the USAXS cell after 3600 Zn ML (1882.8 mC∙cm^−2^) of charge passed. The observed domains are outlined for the corresponding AFM and SEM images in (**e**–**h**). [Reproduction with permission from Keist, J.S.; Hammons, J.A.; Wright, P.K.; Evans, J.W.; Orme, C.A., Electrochim. Acta; published by Elsevier, 2020].

Liu et al. [85] also reported a significant impact of water content on the properties of Zn deposits. More dense and adherent coatings were obtained from imidazolium based ILs in comparison to pyrrolidinium based ILs. The smoothest Zn coating was deposited from the solution with 5%wt H_2_O. A further increase of water content in ILs resulted the coatings deteriorates. Later these authors investigated the influence of anion type in zinc salt on the electrodeposition process of Zn layers [88]. They reported the best properties of Zn coatings obtained in ILs with methylsulfonate anions. Keist et al. [82,87] presented an in-depth analysis of the electrodeposition mechanism of Zn from [BMIm][TfO] with 0.34 mol∙kg^−1^ zinc triflate as a precursor. Zinc was deposited at different applied potentials of −0.4, −0.5, −0.65 V vs. Zn. Application such types of electrolytes leads to obtain crystalline coatings characterized by well-defined hexagonal facets, see Figure 5.

Abbott et al. [89] electrodeposited Zn from ZnCl_2_ dissolved in ChCl based ionic liquids. The authors observed fast nucleation in the urea based liquid but slow bulk growth. In contrast to ChCl:urea application of ChCl:EG leads to obtain slow nucleation and relatively fast bulk growth of Zn film. The mixtures of 1-butylpyrrolidine and ZnCl_2_ examined Pulletikurthi et al. [90] but obtained Zn deposits were metal nanoplates. [BMPyr][DCA] as the medium for electroplating Zn on Mg alloy substrate investigated Deng [91]. In turn, a new type of N-alkylimidazole zinc(II)-containing [Tf_2_N] ionic liquids examined Steichen et al. [92]. Obtained at 90 °C and −100 mA∙cm^−2^ deposits were smooth, crystalline, crack free and compact.

The literature data also provided information on zinc alloys. For instance Zn-Sn films were investigated e.g., Fashu et al. [93]. Due to the fact that tin can also be activated in a cyclotron these types of alloys are unlikely to find application as target materials. The procedures leading to obtain metallic zinc coatings are well explored and elaborated.

### 2.5. Selenium (Se)

Low thermal conductivity, boiling point, and high vapor pressure [94] significantly limit elementary selenium target tolerance to irradiation hence intermetallic compounds are considered as potential materials. As a promising materials NiSe or CoSe are considered suitable [95]. Although both metals can be activated in proton flux, generated isotopes have suitable half-lives, ^59^Ni (T_1/2_ = 76,000 years), ^58^Co (T_1/2_ = 23.7 min). Ellison et al. [95] reported application of CoSe for production of ^76,77,80m^Br.

Elemental selenium was electrodeposited by e.g., Zein El Abedin et al. [96] from ([BMPyr][Tf_2_N]). They obtained a dark deposit at potential of −1.1 V vs. Pt as a QRE. The authors concluded that a single selenium phase was observed at elevated temperatures (100 °C) in contrast to room temperature conditions where amorphous Se was deposited. Saha et al. [97] applied the same IL. The plating procedure required application potential of −1.4 V (vs. Ag│Ag^I^ RE) to glassy carbon electrode. In both cases obtained deposit were characterized by needles, crystals or stars-like aggregates. Electrodeposition of selenium alloys were also of interest. Although due to activation in proton flux (e.g., Cd, Sb, Cu) these alloys cannot be considered as good candidates as selenium alloys targets.

### 2.6. Molybdenum (Mo)

Natural or enriched molybdenum targets are prepared by sintering elemental Mo powder, its melting or direct use of metallic molybdenum foil [98,99] and ref. therein. In the literature one may find also application of Mo compounds, e.g., ^100^Mo_2_C_,_^100^MoS_2_ or as potential target material [100,101].

**Figure 6:**
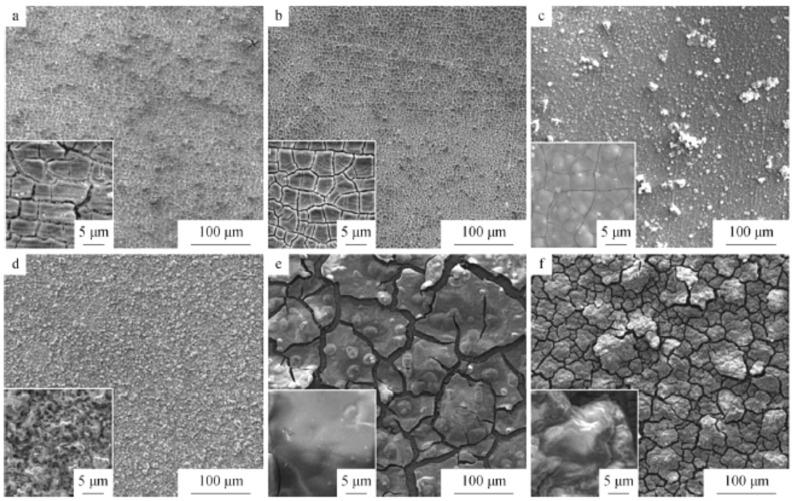
Surface SEM images of Mo layer deposited under different current densities: (**a**) 0.25 mA∙cm^−2^, (**b**) 0.5 mA∙cm^−2^, (**c**) 0.75 mA∙cm^−2^, (**d**) 1.0 mA∙cm^−2^, (**e**) 2.5 mA∙cm^−2^, and (**f**) 5.0 mA∙cm^−2^ in [BMIm][BF4] + EG at 150 °C. [Reproduction with permission from Hu, X.-T.; Qian, J.-G.; Yin, Y.; Li, X.; Li, T.-J.; Li, J., Rare Met. published by Springer, 2018].

The electrochemical properties of molybdenum in aqueous as well in non-aqueous solutions is very complex due to simultaneous chemical reactions in electrochemical reactions. An overview and in-depth analysis of these processes have been presented by Malyshev [102,103]. Nitta et al. [104] electrodeposited Mo from ([EMPyr]Cl)-ZnCl_2_ with 0.9%mol MoCl_5_ and 3%mol KF as additive at 150 °C. They reported smooth, ultrathin (0.2 µm) Mo layers obtained at potential of 0.01 V vs. Zn│Zn^2+^. Hu et al. [105] investigated the process of Mo electrodeposition in ([BMIm][BF4]), with 0.15 M MoCl_5_ at different temperatures, current densities and additive as ethylene glycol (EG). They concluded that in [BMIm][BF4] the electrodeposition of amorphous Mo layers leads to obtain bad adhesive layers with the substrate. The optimal electroplating mixture consists [BMIm][BF4] and EG with the ratio 2:1. Other parameters are temperature at 150 °C; j = 0.75 mA∙cm^−2^. SEM imagines of Mo layers are present in Figure 6. Unfortunately, the deposits are cracked or not smooth.

An earlier work of Gao et al. [106] presented the results of Mo electrodeposition from Li[Tf_2_N]-Cs[Tf_2_N] + MoCl_5_ systems at elevated temperature (150 °C) and j = 0.5 mA∙cm^−2^. Obtained deposits were much more cracked than those aforementioned by Hu et al. [105].

Molybdenum compounds were also electrodeposited from ILs. For instance, Murugesan et al. [107] obtained MoSx deposits from ([MPPip][Tf_2_N]), with molybdenum glycolate and 1,4-butanedithiol as precursors. These layers were obtained at −2V vs. Pt QRE at elevated temperatures (up to 100 °C). In turn, Redman et al. [108] described the electrodeposition of MoS_x_ layers from 1-ethyl-3-methylimidazolium bis(trifluoromethylsulfonyl)imide and MoS_4_^2−^. This layer consisted of densely packed nanoparticles with average grain size of 18 ± 6 nm.

Molybdenum deposits can be obtain from ILs. Obtained layers are not smooth and the composition of the electroplating bath and the electroplating conditions needs to be improved. Moreover, molybdenum sulfides although seems as an attractive alternative to metallic layers, have lower thermal conductivity. From the point of view of their potential application as cyclotron targets the number of cracks in the surface should be reduced.

### 2.7. Cadmium (Cd)

As a target material for ^111^In cyclotron production natural or enriched cadmium with thickness of c.a. 80 µm is widely used [7]. Due to its low melting point (321 °C) elemental cadmium can be initially melted, cut for desired dimension and pressed to obtain appropriate target material properties.

**Figure 7:**
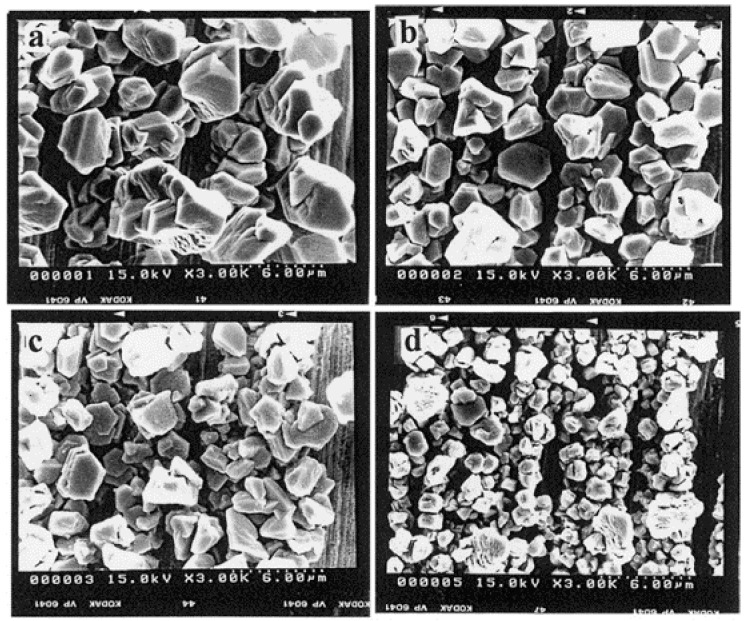
SEM micrographs of cadmium electrodeposits on tungsten wires that were produced in a 37.9 mM Cd(II) in basic [EMIm]Cl/BF_4_ melt at 30°C and the following applied potentials, (**a**) −1.1; (**b**) −1.12; (**c**) −1.14; and (**d**) −1.2 V. [Reproduction with permission from Chen, P.Y.; Sun, I.W., Electrochim. Acta published by Elsevier, 2000].

Potential application of ILs as a solution to form cadmium electrodeposition has been presented in several papers. In 1990 Noël and Osteryoung [109] discussed deposition of cadmium layers from AlCl_3_ and [EMIm]Cl mixtures. In 2000 Chen and Sun [110] have shown the application of [EMIM]Cl/BF_4_ solutions as a medium for electrodeposition of cadmium deposits. This deposit was generated at potentials lower than −1.1 V vs. aluminum wire immersed in a 0.60 mol% AlCl_3_–[EMIm]Cl. They observed that granulation of the deposits decreased as the deposition potential moved more negatively (Figure 7). Discussed process proceeds via 3D progressive nucleation with diffusion-controlled growth of Cd crystals.

In turn, Pan and Freyland [111] demonstrated ultrathin layers of Cd deposited from 1-methyl-3-butylimidazolium chloride–aluminum chloride ionic liquids, in the Under Potential Deposition (UPD) regime. Later Saha et al. [112] electrodeposited cadmium from the mixture of [BMPyr]Cl and [BMPyr][Tf_2_N] with CdCl_2_. These authors obtained sponge like layers on glassy carbon electrodes by applying −2.5 V vs. Ag^+^│Ag^0^ RE.

Cadmium alloys (e.g., CdTe) [113] and its compounds (e.g., CdS) [114] can also be electrodeposited from ILs but their application as cyclotron targets is limited due to activation of co-deposited element (Te, Cu, Zn) or their low thermal conductivities. Characteristic feature of all aforementioned cadmium and cadmium-alloys deposits was their relatively high granulation.

### 2.8. Tellurium (Te)

Various types of enriched tellurium targets are used to production of iodine isotopes. Tellurium powder mixed with aluminum, electrodeposited elemental Te, its alloys or its oxide have proven suitable targets [7]. Unfortunately due to low melting point of tellurium and low sublimation point of radioiodine, some loss of the latter is observed during irradiation of the target. A disadvantage of elemental Te target is also its low thermal conductivity. The procedure for electrodeposition of Te from alkaline solutions are in the literature [115].

Electrodeposition of elemental Te from ionic liquids was of interest of Jeng and Sun [116]. These authors obtained Te deposits from aluminum chloride and 1-methyl-3-ethylimidazolium chloride mixtures applying to the glassy carbon working electrode potential of −0.8 V vs. Al. In a four-electron process [TeCl_6_]^2−^ ions are reduced to Te. This process was specified as nucleation under kinetic control. Unfortunately, obtained deposit was poorly adherent to the substrate. Later Szymczak et al. [117] reported that by using a piperidinium-based ionic liquid mixture [EOPip][Tf_2_N] with [EOPip]Br single crystalline 30–200 nm wires of Te can be electrodeposited. Al-Salman et al. [118] delivered that deposition of Te at −1.2 V from 0.5 M SiCl_4_ + 0.2 M TeCl_4_ dissolved in ([BMPyr][Tf_2_N]) solution leads to obtain the materials with strong tendency toward one-dimensional growth in ILs.

Bartlett et al. [119] electrodeposited tellurium layers onto glassy carbon or titanium nitride electrodes. The experiments were carried out in 0.01M [TBA]_2_[TeCl_6_] dissolved in dichloromethane. To increase the conductivity of the solution [TBA]Cl (0.1M∙dm^−3^) was added. Obtained at potential of −0.4V vs. Ag│AgCl (0.1 mol∙dm^−2^ [TBA]Cl in CH_2_Cl_2_) deposit consisted of homogeneous nanoparticulates characterized by the much better adhesion than In, Sb, Bi or Se films.

**Figure 8:**
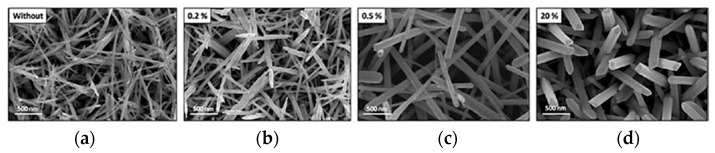
SEM images of Te nanostructures electrodeposited at C1 peak potential (varies from −0.58 V to −0.67 V vs. Ag^I^/Ag) in pure [EOPip][Tf_2_N] (**a**) without and three [EOPip][Tf_2_N]:[EOPip]Br melts (mol%): (**b**) 0.2%, (**c**) 0.5% and (**d**) 20%. [TeCl_4_] = 5 mM. T = 100 °C, Q = 2C/cm^2^, Pt-coated glass slide. [Reproduction with permission from Thiebaud, L.; Legeai, S.; Ghanbaja, J.; Stein N. Electrochim. Acta; published by Elsevier, 2016].

Thiebaud et al. [120] investigated the influence of 1-ethyl-1-octyl-piperidinium bromide ([EOPip]Br) content as additive to 1-ethyl-1-octyl-piperidinium bis(trifluoromethylsulfonyl)imide ([EOPip][Tf_2_N]) on the growth of Te nanowires. The experiments were carried out at 100 °C and Q = 2C∙cm^−2^ of Pt electrode. It has been shown that bromine has a strong impact on the mechanism of tellurium electrodeposition. They stated that [EOPip]Br content electrolyte at level of 0.2 mol% is the most promising for electrodeposition of Te for thermoelectric applications. SEM imagines of typical Te deposits are present in Figure 8.

In turn dos Santos et al. [121] electrodeposited Te layers on gold/fluorine-doped tin oxide film substrate from DES, 1ChCl:2urea and choline chloride-ethylene glycol mixtures (1ChCl:2EG) containing 50 mM of TeCl_4_. The electrodeposition was carried out at elevated temperatures, from 30 up to 60 (or 80) °C. For instance, at potentials equaled −0.04 V and −0.28 V vs. Ag│AgCl for 1ChCl:2EG and 1ChCl:2urea respectively, in initial stage of Te deposition one-dimensional nanostructures were formed. Moreover, the authors stated that the number of spherical particles increases with temperature. An earlier work of Agapescu et al. [122] was also devoted to electrodeposition of Te films from choline chloride based mixtures but this time with oxalic acid (1ChCl:1Ox). Thanks to good solubility of TeO_2_ in such types of solutions, this oxide was used as a precursor. Electrodeposition of Te layers was performed at 60 °C by applying (depending on the procedure) −0.298 V or −0.370 V vs. Ag. These layers were characterized as spheroidal aggregates in a dense arrangement on the surface.

Electrodeposition of Te alloys were also of interest to researchers. Due to elements which may be activated in a cyclotron (e.g., Cd, Sb, Cu) these alloys cannot be considered, as in case of selenium, as good candidates for Te targets. For instance Hsiu and Sun [123] reported electrodeposition of CdTe at elevated temperatures from 1-ethyl-3-methylimidazolium chloride/tetrafluoroborate. In turn, Tsai et al. [124] electrodeposited PbTe alloy from 1-ethyl-3-methylimidazolium tetrafluoroborate ([EMIm][BF_4_]) at constant applied potential at nickel electrodes. Golgovici et al. [125] electrodeposited BiSbTe for thermoelectric devices from choline chloride and malonic acid solutions. SbTe also examined by Mares et al. [126] in ChCl+U systems. Bi_2_Te_3_ investigated in ChCl:Ox mixtures aforementioned Agapescu et al. [122]. Later, Sb_x_Te_y_ or Cu_x_Te_y_ was electrodeposited and characterized by Catrangiu et al. [127] also from choline based mixture. These compounds are considered as semiconductors.

It should be stressed that a characteristic feature of all Te and tellurium-alloys deposits was their high roughness. Almost all authors describe these structures as nanofibers, nanowires, or nanostars. The formation of these structures may be associated with the presence of chemical species adsorbed on the electrode surface which may act as growth one-dimensional preferential agents. From the point of view of potential application as a cyclotron targets Te deposits obtained from ILs characterized by high viscosity are not favorable. Significant changes in morphology of Te films are observed when ILs are dissolved in organic solvents. For these solutions lower surface roughness is reported. It should keep in mind that aforementioned publications are focused on other than medical application, as thermoelectric or semiconductor compounds.

### 2.9. Thallium (Tl) & Indium (In)

Thallium is electrodeposited from alkaline aqueous solutions containing organic compounds, e.g., EDTA [115]. For instance, Liu et al. [128] reported electrodeposition of Tl_2_O_3_ from KOH containing TlAc. An earlier work of Abd El-Halim and Khalil [129] presented the electrodeposited Tl_2_O and Tl_2_O_3_ mixture from sulfate bath. In turn, Tsirlina et al. [130] discussed nucleation mechanism of thallium oxide generation. To our best knowledge no article on the deposition of Tl from ILs solutions has been published. The electrochemistry of its neighbor in group (Indium) is much more explored. One may expect that the chemistry of both elements in this context will reveal a similarity.

Zein El Abedin et al. [96] electrodeposited indium films from 1-butyl-1-methylpyrrolidinium bis(trifluoromethylsulfonyl)amide containing 0.1 M InCl_3_ at a glassy carbon electrode. The procedure required application of −2.2 V vs. Pt as a QRE to working electrode. Other IL was used by Deferm et al. [131]. These authors reported electrodeposition of indium layer from trihexyl(tetradecyl)phosphonium chloride with In^3+^ at −2.0 V vs. Fc│Fc^+^ RE. This procedure leads to obtain spherical droplets. In turn, Alcanfor et al. [132] applied choline chloride (ChCl) and ethylene glycol (EG) (1ChCl:2EG) with 0.05 M InCl_3_ as a electroplating solution. These authors obtained indium nanorods at 80°C and potential of −0.74V vs. Ag│AgCl.

As for tellurium, also for indium its deposits were characterized by high surface roughness. Low melting point of thallium requires to develop its alloys characterized by high values of this parameter. The procedures leading to obtain smooth layers of the latter need to be elaborated.

### 2.10. Uranium (U)

The electroreduction of U(VI) or U(IV) is mainly considered in the literature as a process leading to separation of uranium from other actinides or fission products, not only in ILs but also in other nonaqueous systems [133] and ref. therein. For instance, recent publication of Geran et al. [134] delivered the information on the electrochemical properties of uranium in molten LiCl-LiF salts. In this context the structure of obtained uranium films plays a minor role. Nikitenko et al. [135] and Takao et al. [136] examined electrochemically uranium chloro-complexes in various ILs. In turn Krishna et al. [137] used 1-butyl-3-methylimidazolium chloride ionic liquid with or without fission products (e.g., Pd, Ru) as a medium for examination of the spectroscopic and electrochemical behavior of UO_2_^2+^ ions.

Lopes and Martinot [138] electrodeposited U or UO_2_ from phenanthrene/terbutylammonium tetrafluoroborate [TBA][BF4] mixtures and Cs_2_UCl_6_ and Cs_2_UO_2_Cl_4_ as feed salts. As a cathode material mercury pool (special reservoir for Hg) was used. Giridhar et al. [139] reported possibility of electrodeposition of mixed uranium oxides from 1.1M TBP/[BMIm][Tf_2_N] with UO_2_(NO_3_)_2_ at −2.1V vs. Pd as a QRE. Wanigasekara et al. [140] checked the possibility of application ILs as a medium for UO_2_ electrodeposition. In 2011 Jagadeeswara Rao [141] reported electrodeposition of metallic uranium from the mixture of ([MPPip][Tf_2_N]) with U(IV). These authors reported that at potential of −2.8 V vs. Fc│Fc^+^ metallic uranium was deposited on platinum working electrode. SEM imagines have shown cracked U coatings. Moreover, they observed rapid dissolution of UO_2_ in H[Tf_2_N]. Recent publication of Bhujbal et al. [142] presented nanosized spherical agglomerates of UO_2_ electrodeposited from chloride [N1444]Cl and UO_2_(NO_3_)_2_ solutions. These coatings were obtained on a glassy carbon electrode at −1.06 V vs. Pt QRE.

**Figure 9:**
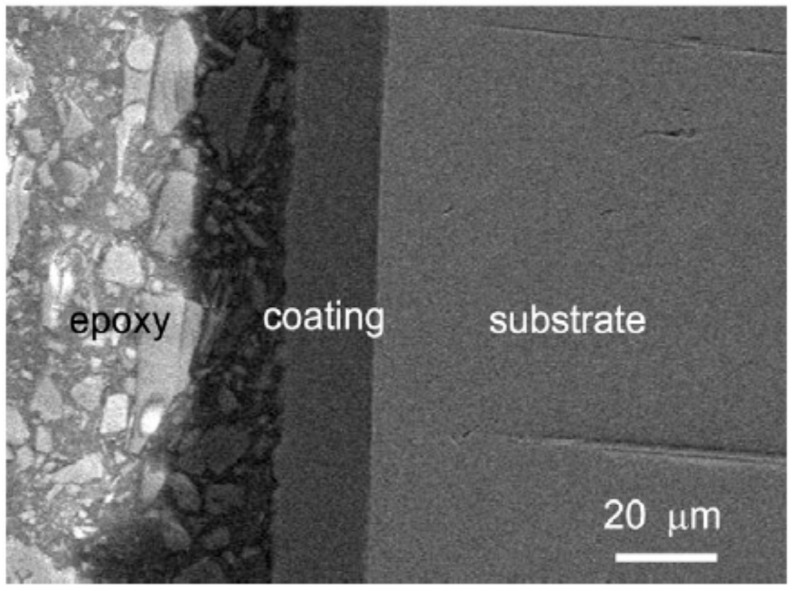
Cross-sectional morphology of Al coating on U: anodically etched in AlCl_3_-[EMIm]Cl (2:1) ionic liquid at 20 mA∙cm^−2^ for 10 min, then electrodeposited in AlCl_3_-[EMIm]Cl (2:1) ionic liquid at 20 mA∙cm^−2^ for 72 C∙cm^−2^ [Reproduction with permission from Jiang, Y.; Ding, J.; Luo, L.; Shi, P.; Wang, X., Surf. Coat. Tech.; published by Elsevier, 2017].

As it was aforementioned, as the targets for reactor production of ^99^Mo, uranium coated with other elements or its alloys are considered. For instance, to improve the corrosion resistance of the material, Jiang et al. [143] electrodeposition thin films of aluminum on uranium (U) substrate from (2AlCl_3_:1[EMIm]Cl) ionic liquid at room temperature. The best results were obtained for current densities of 20 mA∙cm^−2^ (Figure 9). To obtain adhesive Al coatings on uranium substrate, the latter was anodic etched in the pretreatment step. The authors stated that uranium oxides impede the deposition of well-adherent Al surfaces.

The studies on the deposition of Al on the U substrate were continued by Wang and co-workers. In 2018 these authors delivered the information on the growth mechanism of the Al coating on uranium [144]. They have found that Al nano-layer passivates the uranium surface. A characteristic feature of all uranium deposits was their high roughness. Only a few authors delivered the information on metallic films. The vast majority of authors consider uranium oxides as the coatings deposited onto the substrates.

## 3. Application

Electrodeposition of metals can be exploited to provide modified targets with properties that can extend their utilization, ease separation, and increase isotope production. This concept of coupling separation with targetry to permit product isotope collection is an area of interest and has been explore for fission products [145,146]. Most effort has focused on the necessary chemical process, which would separate the fission product and leave the target intact. However, it is understood that target properties, particularly morphology and surface characteristic, are important to the utilization of this technique for development of applications. The ability to achieve product isotope separation from the target without dissolution is a novel application can be facilitated by electroplating. As discussed in the review, the target morphology and structure can be modified through electroplating. The use of substrates with prepared structures and morphology can provide an electroplating platform that produces a target with tailored properties that can be combined with chemistry to removed produced isotopes without target destruction.

Isotope production follows a general workflow that has existed for decades, often with target dissolution after irradiation. The produced isotopes are then separated from the dissolved matrix. In irradiations the vast majority of the target is unreacted. The separation after dissolutions means the minuscule amount of product isotope must be removed from a large amount of target. Depending upon the target, it may be reformed for further isotope production, often with losses and waste generation. The desirability to reuse the target depends upon its properties. If the target is enriched or composed of difficult to obtain material, reuse may be desirable and inconsistent with dissolution followed by separation. An electroplated target, with suitable morphology and material properties, can be used to produce and separate isotopes without dissolution.

The role of radioactive decay and nuclear reactions in initiating chemical reactions is the basis of hot atom chemistry and has been previously explored [147]. The chemical products in nuclear reaction targets are from the chemical effects of the nuclear transformations and the bulk target radiolysis. The energetics from the nuclear activities provides the foundation for initiating reactions that can result in separation without target dissolution. As bond energies are on the order of 10 eV, and nuclear reactions are keV or greater, the direct reaction of daughter recoil can provide sufficient energy for bond breaking as a first step to chemical reactions. Recoil based separations have long been used in production of the heaviest elements in atom-at-a-time reactions, where the product nuclei relies upon the nuclear reaction for the first separation [148,149].

For radiopharmaceuticals, nuclear reactions have been examined as a route to increase an isotope specific activity for reactions that included non-active isotopes as the initial target [147]. An example is the production reaction ^127^I(n,γ)^128^I. The ability to achieve isotopic separations through nuclear reactions and subsequent decay was initially demonstrated with ethyl iodide [150]. The ^127^I on the ethyl iodide is separated from the recoiled, anionic ^128^I^−^. The bulk of these reactions involve molecular or gas phase species. The ability to achieve separation from bulk solid phase has been less explored, but some trends are observable. This method is effective with alpha decay, less so with beta and gamma decay. Nuclear reactions were shown to be as effective as alpha decay.

Coupling targetry with separations through product nuclei recoil from the nuclear reaction has been demonstrated with U fission with UO_2_ and U-metal-organic framework as targets [151,152]. From this work refractory uranium oxide particles combined with an easily dissolved salt demonstrated effective recovery of select fission products. The recoil of fission products is around 10 µm [153]. For electroplated uranium targets, the recoiling fission products can separate from the target matrix. Exploiting differences between uranium and the fission products. Electroplated uranium targets, ideally the metal but also the oxide, and be coupled with selected separation routes [145,146] for targetry coupled separations for molybdenum.

Clearly fission, with decay energies around 200 MeV, provides sufficient energy for exploitation of the nuclear decay in separation. Lower nuclear reaction or decay energies can also be suitable to drive the first step of the separation process. Studies on nuclear waste forms provide data on expected chemical reaction trends due to isotope decay within a matrix. Chemical reactions in beta emitter containing waste forms show decay can initiate reactions for certain isotopes [154]. The trend favors reactions for those isotopes with shorter half-lives. Other studies examining host compound found structural changes due to decay could result in formation of instabilities [155]. These instabilities could be the basis for separations. The structure of the target compound containing the produced isotope can be tuned to optimize chemical reactions. In this way, using ionic liquid based electroplating, a target for irradiation can be constructed to permit chemical separations due to the nuclear reaction and subsequent decay, even in conditions without this high energy from fission. This provides a means to apply this electroplating method to the production of a range of diagnostic isotopes.

## 4. Conclusions

From the point of view of potential application of the electrodeposited layers of discussed elements as cyclotron targets two parameters are critical. Obtained coatings must be smooth and adherent as much as it possible to the substrate. Resulting morphologies from the substrate and electrochemistry electroplating can be used to supplement isotope production.

Only two from aforementioned elements exist in one-isotopic form, scandium and yttrium. The targets produced from the other elements is the most often enriched in desired isotope. This situation requires high electrodeposition yields. From this point of view available in the literature electroplating protocols need to be checked and adapted to the target productions.

Electroplating baths are well known for zinc and nickel. Electrodeposition of calcium as well as strontium creates serious experimental problems although recent publication shows a possibility coating of metallic Ca from IL. The literature devoted to characterization of scandium and yttrium is poor. One may expect that as for lanthanides these elements can be deposited from ionic liquids. In case of tellurium its films can be obtained. Unfortunately, due to its low meting point and low thermal conductivity, a new alloys need developed. Similar situation is observed for cadmium. Metallic molybdenum layers are difficult to obtain both in water and organic solvents due to various sides chemical reactions accompanying the deposition process. Only a few publications are devoted to electrodeposition of metallic uranium. Electrodeposition of this element is also difficult. Application of ILs as a medium leads more often to electrodeposition of its oxides.

Despite the emerging difficulties, it seems that ionic liquids as a reaction medium for electroplating of desired elements constitute an interesting alternative to existing methods of cyclotron target preparation.

## Figures and Tables

**Table 1:** Characterization of selected cyclotron and reactor radioisotopes for nuclear imagining [3,4,5,6,7].

Production	Radio-Nuclide	Main Decay Scheme	Half Life	Nuclear Reaction	Target Isotope	m_p_/°C	Thermal Cond./ W∙m^−1^∙K^−1^	Abund. of Target Isotope/%
cyclotron	^44^Sc	β^+^	3.93 h	^44^Ca(p,n)^44^Sc	^44^Ca	842	200	2.086
	^45^Ti	β^+^	3.1 h	^45^Sc(p,n)^45^Ti	^45^Sc	1539	16	100
	^55^Co	β^+^	17.5 h	^58^Ni(p,α)^55^Co	^55^Ni	1455	91	68.0769
	^60^Cu	β^+^	23.7 min	^60^Ni(p,n)^60^Cu	^60^Ni			26.2231
	^61^Cu	β^+^	3.3 h	^61^Ni(p,n)^61^Cu	^61^Ni			1.1399
	^64^Cu	β^+^	12.7 h	^64^Ni(p,n)^64^Cu	^64^Ni			0.9256
	^68^Ga	β^+^	67.7 min	^68^Zn(p,n)^68^Ga	^68^Zn	419.5	120	18.5
	^76^Br	β^+^	16.2 h	^76^Se(p,n)^76^Cu	^76^Se	217	0.52	9.37
	^86^Y	β^+^	14.7 h	^86^Sr(p,n)^86^Y	^86^Sr	770	35	9.86
	^89^Zr	β^+^	3.27 days	^89^Y(p,n)^89^Zr	^89^Y	1523	17	100
	^94m^Tc	IT	52 min	^94^Mo(p,n)^94m^Tc	^94^Mo	2617	139	9.23
	^99m^Tc	IT	6.02 h	^100^Mo(p,2n)^99m^Tc	^100^Mo			14.77
	^111^In	β^-^	2.8 days	^111^Cd(p,n)^111^In	^111^Cd	321	97	12.80
	^124^I	β^+^	1.18 days	^124^Te(p,n)^124^I	^124^Te	450	3	4.74
	^201^Tl	EC	73 h	^203^Tl(p,3n)^201^Pb→^201^Tl	^203^Tl	304	46	29.52
nuclear reactor	^99^Mo→ ^99m^Tc	IT	66h→6.02 h	^235^U(n,xn) SF (incl. ^99^Mo→^99m^Tc)	^235^U	1132	27	varies ^1^
	^131^I	β^-^	8 days	^130^Te(n,γ)^131^Te→^131^I	^130^Te	321	3	12.80

^1^ depends on the technology, the enrichment can be high (above 80%) or low (c.a. 3%).

**Table 2:** Atomic densities of the elements in metals and their oxides.

Element	Atomic Density of the Element in: (10^23^ at.∙cm^−3^)		at. Ratio Metal: Metal Oxide
	Metal	Metal Oxide	
Ca	0.233	0.359 (CaO)	1.18
Sc	0.399	0.339 (Sc_2_O_3_)	1.18
Ni	0.914	0.538 (NiO)	1.70
Zn	0.657	0.415 (ZnO)	1.58
Se	0.367_(grey)_	0.215 (SeO_2_)	1.71
Sr	0.181	0.273 (SrO)	0.66
Y	0.302	0.266 (Y_2_O_3_)	1.14
Mo	0.645	0.197 (MoO_3_)	3.27
U	0.483	0.244 (UO_2_)	1.98
Te	0.294	0.228 (TeO_2tetra_)	1.29

**Table 3:** Solubility of metal oxides (in ppm) in DES [55,56].

Oxide	(2:1) Malonic Acid/ Choline Chloride	(2:1) Urea/ Choline Chloride	Temp./°C
TiO_2_	4	0.5	50
CoO	3626	13.6	
Co_3_O_4_	5992	30	
CuO	14,008	4.8	
Cu_2_O	18,337	219	
NiO	151	5	
ZnO	16,217	1894	
MnO_2_	114	0.6	
PbO_2_		9157	60
CuO		470	
NiO		325	

## Abbreviations

ChCl	Choline chloride
EG	Ethylene glycol
Mal	Malonic acid
Lev	Levulinic acid
Phenylac	Phenylacetic acid
Phenylprop	3-Phenylpropionic acid
Glyce	Glycerol
Gluc	D-(+)-Glucose
TBP	tri-n-butylphosphate
[BMIm][DBP]	1-butyl-3-methylimidazolium dibutylphosphate
[BMIm][HSO_4_]	1-butyl-3-methylimidazolium hydrogen sulfate
[BMIm][TfO]	1-butyl-3-methylimidazolium trifluoromethanesulfonate
[BMIm][BF_4_]	1-butyl-3-methylimidazolium tetrafluoroborate
[EMIm][BF_4_]	1-ethyl-3-methylimidazolium tetrafluoroborate
[P2225][Tf_2_N]	triethylopentylophosphonium bis(trifluoromethylsulfonyl)amide
[P66614][Tf_2_N]	trihexyltetradecylphosphonium bis(trifluoromethylsulfonyl)amide
[BMPyr][Tf_2_N]	1-butyl-1-methylpyrrolidinium bis(trifluoromethylsulfonyl)amide
[EMPyr]Cl	N-ethyl-N-methyl-pyrrolidinium chloride
[BMPyr][DCA]	N-butyl-N-methyl-pyrrolidinium dicyanamide
[BMPyr]Cl	1-butyl-1-methylpyrrolidinium chloride
[EMIm][DCA]	1-ethyl-3-methylimidazolium dicyanamide
[EMIm]Cl	1-Ethyl-3-methylimidazolium chloride
[N1444]Cl	tri-n-butylmethylammonium chloride
[TBA][BF4]	terbutylammonium tetrafluoroborate
[TBA]_2_[TeCl_6_]	tetrabutylammonium hexachlorotellurate(IV)
[TBA]Cl	tetrabutylammonium chloride
[TMA]Cl	tetramethylammonium chloride
[EOPip]Br	1-ethyl-1-octyl-piperidinium bromide
[EOPip][Tf_2_N]	1-ethyl-1-octyl-piperidinium bis(trifluoromethylsulfonyl)imide
[MPPip][Tf_2_N]	1-methyl-1-propylpiperidinium bis(trifluoromethanesulfonyl)imide

## References

[1] Mettler F.A., Bhargavan M., Faulkner K., Gilley D.B., Gray J.E., Ibbott G.S., Lipoti J.A., Mahesh M., McCrohan J.L., Stabin M.G. (2009). Radiologic and nuclear medicine studies in the United States and worldwide: Frequency, radiation dose, and comparison with other radiation sources—1950–2007. Radiology.

[2] Martin J.E. (2006). Physics for Radiation Protection.

[3] IAEA (2017). Cyclotron based production of technetium-99m. IAEA Radioisotopes and Radiopharmaceuticals Reports.

[4] Costa P., Metello L.F., Alves F., Duarte N.M. (2018). Cyclotron production of unconventional radionuclides for PET imaging: The example of Titanium-45 and its applications. Instruments.

[5] (2020). Thermal Conductivity of the Elements. https://www.periodic-table.org/thermal-conductivity-of-chemical-elements/.

[6] Baum E.M., Ernesti M.C., Knox H.D., Miller T.R., Watson A.M. (2010). Chart of the Nuclides.

[7] IAEA (2009). Cyclotron Produced Radionuclides: Physical Characteristics and Production Methods.

[8] Bechtold V. (1996). Isotope Production with Cyclotrons. https://cds.cern.ch/record/399436/files/p329.pdf.

[9] Taghilo M., Kakavand T., Rajabifar S., Sarabadani P. (2012). Cyclotron production of 89Zr: A potent radionuclide for positron emission tomography. Int. J. Phys. Sci..

[10] Yiğit M. (2018). Model-based cross section calculations on production of ^43,34^Sc, ^45^Ti, ^51^Cr, ^54^Mn, and ^55^Fe radioisotopes. Nucl. Sci. Tech..

[11] Krzysztoszek G., Jaroszewicz J., Pytel K. Irradiations of HEU targets in Maria rr for Mo-99 production. Proceedings of the IAEA Technical Meeting on Commercial Products and Services of Research Reactors, (IAEA-TM-38728).

[12] Krzysztoszek G. The characteristics and irradiation capabilities of MARIA research reactor in NCBJ Świerk. Proceedings of the 2nd International Workshop Irradiation of Nuclear Materials: Flux and Dose Effects, CEA–INSTN Cadarache.

[13] Jaroszewicz J., Marcinkiewicz Z., Pytel K. (2014). Production of fission product 99Mo using high-enriched uranium plates in Polish nuclear research reactor MARIA: Technology and neutronic analysis. Nukleonika.

[14] Raposio R., Thorogood G., Czerwinski K., Rozenfeld A. (2019). Development of LEU-based targets for radiopharmaceutical manufacturing: A review. Appl. Rad. Isotop..

[15] Wong R., Gelbart W.Z., Stevenson N.R., Wong A. (2000). Investigation of thermal-mechanical properties of solid targets for radioisotopes production. Isotope Production and Applications in the 21st Century.

[16] Gofryk K., Du S., Stanek C.R., Lashley J.C., Liu X.-Y., Schulze R.K., Smith J.L., Safarik D.J., Byler D.D., McClellan K.J. (2014). Anisotropic thermal conductivity in uranium dioxide. Nat. Commun..

[17] Ortega L.H., Blamer B.M., Stern K., Vollmer J., McDeavitt S.M. (2020). Thermal conductivity of uranium metal and uranium-zirconium alloys fabricated via powder metallurgy. J. Nucl. Mat..

[18] Biswal A., Panda P.K., Acharya A.N., Mohapatra S., Swain N., Tripathy B.C., Jiang Z.-T., Sundaram M.M. (2020). Role of additives in electrochemical deposition of ternary metal oxide microspheres for supercapacitor applications. ACS Omega.

[19] Van Den Bosch R., De Goeij J.J.M., Van Der Heide J.A., Tertoolen J.F.W., Theelen H.M.J., Zegers C. (1977). A new approach to target chemistry for the iodine-123 production via the ^124^Te(p, 2n)123I reaction. Int. J. Appl. Radiat. Isot..

[20] Rard J.A., Rand M.H., Anderegg G. (1999). Chemical Thermodynamics of Technetium.

[21] Inzelt G., Bard A.J., Stratmann M. (2007). Standard, formal, and other characteristic potentials of selected electrode potentials. Encyclopedia of Electrochemistry.

[22] Oh J.-S., Warwick P.E., Croudace I.W., Lee S.-H. (2014). Evaluation of three electrodeposition procedures for uranium, plutonium and americium. Appl. Rad. Isotop..

[23] Krmpotića M., Rožmarića M., Benedik L. (2018). Investigation of key factors in preparation of alpha sources by electrodeposition. Appl. Rad. Isotop..

[24] Kumbhar P.P., Lokhande C.D. (1995). Electrodeposition of yttrium from a nonaqueous bath. Met. Finish..

[25] Ondono-Castillo S., Fuertes A., Perez F., Gomez-Romero P., Casan-Pastor N. (1995). Superconducting YBa2Cu3O7-δ coatings by simultaneous electrodeposition of Y, Ba, and Cu in the presence of Cyanide. Chem. Mater..

[26] Shirasaki K., Yamamura T., Herai T., Shiokawa Y. (2006). Electrodeposition of uranium in dimethyl sulfoxide and its inhibition by acetylacetone as studied by EQCM. J. Alloys Compd..

[27] Audrieth L.F., Nelson H.W. (1931). Electrodeposition of metals from nonaqueous solvents. Chem. Rev..

[28] Panzeri G., Muller D., Accogli A., Gibertini E., Mauri E., Rossi F., Nobili L., Magagnin L. (2019). Zinc electrodeposition from a chloride-free non-aqueous solution based on ethylene glycol and acetate salts. Electrochim. Acta.

[29] Ta K., See K.A., Gewirth A.A. (2018). Elucidating Zn and Mg electrodeposition mechanisms in nonaqueous electrolytes for next-generation metal batteries. J. Phys. Chem. C.

[30] Inman D., Mamantov G., Marassi R. (1987). Electrodeposition from molten salts. Molten Salt Chemistry. An lntroduction and Selected Applications.

[31] Kerridge D.H., Polyakov E.G. (1998). Refractory metals in molten salts. Their chemistry, electrochemistry and technology. NATO ASI Series, 3. High. Technology.

[32] Shtefanyuk Y., Mann V., Pingin V., Vinogradov D., Zaikov Y., Tkacheva O., Nikolaev A., Suzdaltsev A., Hyland M. (2015). Production of Al-Sc alloy by electrolysis of cryolite-scandium oxide melts. Light Metals.

[33] Gu Y., Liu J., Qu S., Deng Y., Han X., Hu W., Zhong C. (2017). Electrodeposition of alloys and compounds from high-temperature molten salts. J. Alloys Compd..

[34] Castrillejo Y., Hernández P., Rodriguez J.A., Vega M., Barrado E. (2012). Electrochemistry of scandium in the eutectic LiCl–KCl. Electrochim. Acta.

[35] Ohno H. (2005). Electrochemical Aspects of Ionic Liquids.

[36] Kosmulski M., Gustafsson J., Rosenholm J.B. (2004). Thermal stability of low temperature ionic liquids revisited. Thermochim. Acta.

[37] Maton C., De Vos N., Stevens C.V. (2013). Ionic liquid thermal stabilities: Decomposition mechanisms and analysis tools. Chem. Soc. Rev..

[38] Delgado-Mellado N., Larriba M., Navarro P., Rigual V., Ayuso M., García J., Rodríguez F. (2018). Thermal stability of choline chloride deep eutectic solvents by TGA/FTIR-ATR analysis. J. Mol. Liq..

[39] Tang B., Bi W., Tian M., Row K.H. (2012). Application of ionic liquid for extraction and separation of bioactive compounds from plants. Review. J. Chromatogr. B.

[40] Gonzaleza A.S.B., Franciscoa M., Jimenoa G., Lago García de Diosb S., Kroona M.C. (2013). Liquid–liquid equilibrium data for the systems {LTTM + benzene + hexane} and {LTTM + ethyl acetate + hexane} at different temperatures and atmospheric pressure. Fluid Phase Equil..

[41] Li J., Han Z., Zou Y., Yu B. (2015). Efficient extraction of major catechins in *Camellia sinensis* leaves using green choline chloride-based deep eutectic solvents. RSC Adv..

[42] Li X., Hou M., Zhang Z., Han B., Yang G., Wanga X., Zou L. (2008). Absorption of CO_2_ by ionic liquid/polyethylene glycol mixture and the thermodynamic parameters. Green Chem..

[43] Patino J., Gutierrez M.C., Carriazo D., Ania C.O., Parra J.B., Ferrera M.L., del Monte F. (2012). Deep eutectic assisted synthesis of carbon adsorbents highly suitable for low-pressure separation of CO_2_–CH_4_ gas mixtures. Energy Environ. Sci..

[44] Cui G., Lv M., Yang D. (2019). Efficient CO_2_ absorption by azolide-based deep eutectic solvents. Chem. Commun..

[45] Xie Y., Liu G., Nie H., Yu F., Xing X., Cui H. (2020). Energy analysis of physical absorption and chemical absorption of CO_2_ in ionic liquids. Energy Technol..

[46] Liao H.G., Jiang Y.X., Zhou Z.Y., Chen S.P., Sun S.G. (2008). Shape-Controlled synthesis of gold nanoparticles in deep eutectic solvents for studies of structure–functionality relationships in electrocatalysis. Angew. Chem. Int. Ed..

[47] Kuhn B.L., Paveglio G.C., Silvestri S., Muller E.I., Enders M.S.P., Martins M.A.P., Zanatta N., Bonacorso H.G., Radke C., Frizzo C.P. (2019). TiO_2_ nanoparticles coated with deep eutecticsolvents: Characterization and effect on photodegradation of organic dyes. New J. Chem..

[48] Verma C., Ebenso E.E., Quraishi M.A. (2019). Transition metal nanoparticles in ionic liquids: Synthesis and stabilization. Review. J. Mol. Liq..

[49] Javed F., Ullah F., Zakaria M.R., Akil H.M. (2018). An approach to classification and hi-tech applications of room temperature ionic liquids (RTILs): A review. J. Mol. Liq..

[50] Pająk M., Hubkowska K., Czerwiński A. (2020). Hydrogen sorption capacity as a tunable parameter in aprotic ionic liquids. Electrochem. Commun..

[51] Jónsson E. (2020). Ionic liquids as electrolytes for energy storage applications—A modelling perspective. Energy Stor. Mater..

[52] Vekariya R.L. (2017). A review of ionic liquids: Applications towards catalytic organic transformations. J. Mol. Liq..

[53] Juneidi I., Hayyana M., Hashima M.A. (2018). Intensification of biotransformations using deep eutectic solvents: Overview and outlook. Process. Biochem..

[54] Liu F., Deng Y., Han X., Hu W., Zhong C. (2016). Electrodeposition of metals and alloys from ionic liquids. J. Alloys Compd..

[55] Abbott A.P., Capper G., Davies D.L., Rasheed R.K., Shikotra P. (2005). Selective extraction of metals from mixed oxide matrixes using choline-based ionic liquids. Inorg. Chem..

[56] Abbott A.P., Capper G., Davies D.L., McKenzie K.J., Obi S.U. (2006). Solubility of metal oxides deep eutectic solvents based on choline chloride. J. Chem. Eng. Data.

[57] Sadeghi M., Aboudzadeh M., Zali A., Mirzaii M., Bolourinovin F. (2009). Radiochemical studies relevant to ^86^Y production via ^86^Sr(p,n)^86^Y forPET imaging. Appl. Rad. Isotop..

[58] Ta K., Zhang R., Shin M., Rooney R.T., Neumann E.K., Gewirth A.A. (2019). Understanding Ca electrodeposition and speciation processes in nonaqueous electrolytes for next-generation Ca-ion batteries. ACS Appl. Mater. Interfaces.

[59] Hahn N.T., Self J., Seguin T.J., Driscoll D.M., Rodriguez M.A., Balasubramanian M., Persson K.A., Zavadil K.R. (2020). The critical role of configurational flexibility in facilitating reversible reactive metal deposition from borohydride solutions. J. Mater. Chem. A.

[60] Biria S., Pathreeker S., Genier F.S., Li H., Hosein I.D. (2020). Plating and stripping calcium at room temperature in an ionicliquid electrolyte. ACS Appl. Energy Mater..

[61] Minakshi M., Mitchell D.R.G., Munnangi A.R., Barlow A.J., Fichtner M. (2018). New insights into the electrochemistry of magnesium molybdate hierarchical architectures for high performance sodium devices. Nanoscale.

[62] Chen P.-Y., Hussey C.L. (2005). Electrochemistry of ionophore-coordinated Cs and Sr ions in the tri-1-butylmethylammonium bis((trifluoromethyl)sulfonyl)imide ionic liquid. Electrochim. Acta.

[63] Chen P.-Y. (2007). The assessment of removing strontium and cesium cationsfrom aqueous solutions based on the combined methods of ionic liquid extraction and electrodeposition. Electrochim. Acta.

[64] Hori S., Hirano M., Ohta R. (2017). Electrodeposition of strontium apatite nanorod arrays and their cel Compatibility. Ceram. Inter..

[65] Ramos J.P. (2020). Thick solid targets for the production and online release of radioisotopes: The importance of the material characteristics—A review. Nucl. Instrum. Methods Phys. Res. Sect. B Beam Interactions Mater. Atoms.

[66] Zhitomirsky I., Petric A. (2000). Electrochemical deposition of yttrium oxide. J. Mater. Chem..

[67] Glukhov L.M., Greish A.A., Kustov L.M. (2010). Electrodeposition of rare Earth metals Y, Gd, Yb in ionic liquids. Russ. J. Phys. Chem. A.

[68] Matsumiya M., Chen J. (2016). Electrodeposition of rare Earth metal in ionic liquids. Application of Ionic Liquids on Rare Earth Green Separation and Utilization.

[69] Sanchez-Cupido L., Pringle J.M., Siriwardana A.L., Unzurrunzaga A., Hilder M., Forsyth M., Pozo-Gonzalo C. (2019). Water-Facilitated electrodeposition of neodymium in a phosphonium-based ionic liquid. J. Phys. Chem. Lett..

[70] Zhu Y.-L., Kozuma Y., Katayama Y., Miura T. (2009). Electrochemical behavior of Ni(II)/Ni in a hydrophobic amide-type room-temperature ionic liquid. Electrochim. Acta.

[71] Florea A., Anicai L., Costovici S., Golgovicic F., Visanc T. (2010). Ni and Ni alloy coatings electrodeposited from choline chloride-based ionic liquids–Electrochemical synthesis and characterization. Surf. Interface Anal..

[72] Cherigui E.A.M., Sentosun K., Bouckenooge P., Vanrompay H., Bals S., Terryn H., Ustarroz J. (2017). Comprehensive study of the electrodeposition of nickel nanostructures from deep eutectic solvents: Self-limiting growth by electrolysis of residual water. J. Phys. Chem. C.

[73] Sebastian P., Giannotti M.I., Gόmez E., Feliu J.M. (2018). Surface sensitive nickel electrodeposition in deep eutectic solvent. ACS Appl. Energy Mater..

[74] Sun C., Zeng J., Lei H., Yang W., Zhang Q. (2019). Direct electrodeposition of phosphorus-doped nickel superstructures from choline chloride−ethylene glycol deep eutectic solvent for enhanced hydrogen evolution catalysis. ACS Sustain. Chem. Eng..

[75] Zheng Y., Zhou X., Luo Y., Yu P. (2020). Electrodeposition of nickel in air-and water-sTable 1-butyl-3-methylimidazolium dibutylphosphate ionic liquid. RSC Adv..

[76] Ash B., Nalajala V.S., Popuri A.K., Subbaiah T., Minakshi M. (2020). Perspectives on nickel hydroxide electrodes suitablefor rechargeable batteries: Electrolytic vs. chemicalsynthesis routes. Nanomaterials.

[77] Du C., Zhao B., Chen X.-B., Birbilis N., Yang H. (2016). Effect of water presence on choline chloride-2urea ionic liquid and coating platings from the hydrated ionic liquid. Sci. Rep..

[78] Lukaczynska M., Cherigui E.A.M., Ceglia A., Van Den Bergh K., De Strycker J., Terryn H., Ustarroz J. (2019). Influence of water content and applied potential on the electrodeposition of Ni coatings from deep eutectic solvents. Electrochim. Acta.

[79] Bernasconi R., Magagnin L. (2017). Electrodeposition of nickel from DES on aluminum for corrosion protection. Surf. Eng..

[80] Gong K., Hua Y.-X., Xu C.-Y., Zhang Q.-B., Li Y., Ru J.-J., Jie Y.-F. (2015). Electrodeposition behavior of bright nickel in air and water-stable betaine·HClethylene glycol ionic liquid. Trans. Nonferrous Met. Soc. China.

[81] IAEA (2019). Gallium-68 Cyclotron Production.

[82] Keist J.S., Hammons J.A., Wright P.K., Evans J.W., Orme C.A. (2020). Coupling in situ atomic force microscopy (AFM) and ultra-small-angle X-ray scattering (USAXS) to study the evolution of zinc morphology during electrodeposition within an imidazolium based ionic liquid electrolyte. Electrochim. Acta.

[83] Borissov D., Pareek A., Renner F.U., Rohwerder M. (2010). Electrodeposition of Zn and Au–Zn alloys at low temperature in an ionic liquid. Phys. Chem. Chem. Phys..

[84] Simons T.J., Torriero A.A.J., Howlett P.C., MacFarlane D.R., Forsyth M. (2012). High current density, efficient cycling of Zn^2+^ in 1-ethyl-3-methylimidazolium dicyanamide ionic liquid: The effect of Zn^2+^ salt and water concentration. Electrochem. Commun..

[85] Liu Z., El Abedin S.Z., Endres F. (2013). Electrodeposition of zinc films from ionic liquids and ionic liquid/water mixtures. Electrochim. Acta.

[86] Xu M., Ivey D.G., Qu W., Xie Z., Dy E., Yuanb X.Z. (2014). Zn/Zn(II) redox kinetics and Zn deposit morphology in water added ionic liquids with bis(trifluoromethanesulfonyl)imide anions. J. Electrochem. Soc..

[87] Keist J.S., Orme C.A., Wright P.K., Evans J.W. (2015). An in situ AFM study of the evolution of surface roughness for zinc electrodeposition within an imidazolium based ionic liquid electrolyte. Electrochim. Acta.

[88] Liu Z., Cui T., Lu T., Ghazvini M.S., Endres F. (2016). Anion effects on the solid/ionic liquid interface and the electrodeposition of zinc. J. Phys. Chem. C.

[89] Abbott A.P., Barron J.C., Ryder K.S. (2009). Electrolytic deposition of Zn coatings from ionic liquids based on choline chloride. T I Met. Finish.

[90] Pulletikurthi G., Ghazvini M.S., Cui T., Borisenko N., Carstens T., Borodin A., Endres F. (2017). Electrodeposition of zinc nanoplates from an ionic liquid composed of 1-butylpyrrolidine and ZnCl_2_: Electrochemical, in situ AFM and spectroscopic studies. Dalton Trans..

[91] Denga M.J., Linb P.C., Changc J.K., Chena J.M., Lua K.T. (2011). Electrochemistry of Zn(II)/Zn on Mg alloy from the N-butyl-N-methylpyrrolidinium dicyanamide ionic liquid. Electrochim. Acta.

[92] Steichen M., Brooks N.R., Meervelt L.V., Fransaerc J., Binnemans K. (2014). Homoleptic and heteroleptic N-alkylimidazole zinc(II)-containing ionic liquids for high current density electrodeposition. Dalton Trans..

[93] Fashu S., Gu C.D., Zhang J.L., Bai W.Q., Wang X.L., Tu J.P. (2015). Electrodeposition and characterization of Zn–Sn alloy coatings from a deep eutectic solvent based on choline chloride for corrosion protection. Surf. Interface Anal..

[94] Baker E.H. (1968). The vapour pressure and resistivity of selenium at high temperatures. J. Chem. Soc. A.

[95] Ellison P.A., Olson A.P., Barnhart T.E., Hoffman S.L.V., Reilly S.W., Makvandi M., Bartels J.L., Murali D., DeJesus O.T., Lapi S.E. (2020). Improved production of 76Br, 77Br and 80mBr via CoSe cyclotron targets and vertical dry distillation. Nucl. Med. Biol..

[96] Zein El Abedin S.Z., Saad A.Y., Farag H.K., Borisenko N., Liu Q.X., Endres F. (2007). Electrodeposition of selenium, indium and copper in an air-and water-stable ionic liquid at variable temperatures. Electrochim. Acta.

[97] Saha S., Tachikawa N., Yoshii K., Katayama Y. (2016). Electrodeposition of selenium in a hydrophobic room-temperature ionic liquid. J. Electrochem. Soc..

[98] Lebeda O., Pruszyński M. (2010). New measurement of excitation functions for (p,x) reactions on ^nat^Mo with special regard to the formation of ^95m^Tc, ^96m+g^Tc, ^99m^Tc and ^99^Mo. Appl. Radiat. Isot..

[99] Cieszykowska I., Janiak T., Barcikowski T., Mielcarski M., Mikołajczak R., Choiński J., Barlak M., Kurpaska Ł. (2017). Manufacturing and characterization of molybdenum pellets used as targets for ^99m^Tc production in cyclotron. Appl. Rad. Isotop..

[100] Richards V.N., Mebrahtu E., Lapi S.E. (2013). Cyclotron production of ^99m^Tc using ^100^Mo_2_C targets. Nucl. Med. Biol..

[101] Gott M.D., Hayes C.R., Wycoff D.E., Balkin E.R., Smith B.E., Pauzauskie P.J., Fassbender M.E., Cutler C.S., Ketring A.R., Wilbur D.S. (2016). Accelerator-based production of the 99mTc-186Re diagnostic therapeutic pair using metal disulfide targets (MoS_2_, WS_2_, OsS_2_). Appl. Rad. Isotop..

[102] Malyshev V.V. (2007). Theoretical foundations and practical realization of molybdenum electrodeposition in ionic melts. Theor. Found. Chem. Eng..

[103] Malyshev V.V. (2009). Mechanisms of electroreduction and electroplating of VI-A group metal coatings from ionic melts. Prot. Met. Phys. Chem. Surf..

[104] Nitta K., Majima M., Inazawa S., Kitagawa K., Nohira T., Hagiwara R. (2010). Electrodeposition of molybdenum from molten salt. SEI Tech. Rev..

[105] Hu X.-T., Qian J.-G., Yin Y., Li X., Li T.-J., Li J. (2018). Approaches to electrodeposit molybdenum from ionic liquid. Rare Met..

[106] Gao B.L., Wang Z.W., Nohira T., Hagiwara R. (2011). Electroreduction of MoCl5 in room temperature ionic liquid at 150 °C. Rare Met. Mater. Eng..

[107] Murugesan S., Akkineni A., Chou B.P., Glaz M.S., Vanden Bout D.A., Stevenson K.J. (2013). Room temperature electrodeposition of molybdenum sulfide for catalytic and photoluminescence applications. ACS Nano.

[108] Redman D.W., Rose M.J., Stevenson K.J. (2017). Electrodeposition of amorphous molybdenum chalcogenides from ionic liquids and their activity for the hydrogen evolution reaction. Langmuir.

[109] Noël M.A.M., Osteryoung R.A. (1990). Use of metal chlorides to buffer neutral ambient temperature molten salts. J. Electroanal. Chem. Interf. Electrochem..

[110] Chen P.-Y., Sun I.-W. (2000). Electrochemistry of Cd(II) in the basic 1-ethyl-3-methylimidazolium chloride/tetrafluoroborate room temperature molten salt. Electrochim. Acta.

[111] Pan G.-B., Freyland W. (2007). In situ STM investigation of spinodal decomposition and surface alloying during underpotential deposition of Cd on Au(111) from an ionic liquid. Phys. Chem..

[112] Saha S., Tachikawa N., Yoshii K., Serizawa N., Katayama Y. (2018). Electrodeposition of Cadmium from Lewis basic hydrophobic room-temperature ionic liquid. Electrochem.

[113] Waldiya M., Bhagat D., Mukhopadhyay I. (2020). Photoelectrochemical study of electrochemically synthesized CdTe thin films from acetate-anion based ionic liquid bath. Electrochim. Acta.

[114] Dale P.J., Samantilleke A.P., Shivagan D.D., Peter L.M. (2007). Synthesis of cadmium and zinc semiconductor compounds from an ionic liquid containing choline chloride and urea. Thin Sol. Film..

[115] IAEA (2004). Standardized High Current Solid Targets for Cyclotron Production of Diagnostic and Therapeutic Radionuclides.

[116] Jeng E.G.S., Sun I.W. (1997). Electrochemistry of tellurium(IV) in the basic aluminum chloride-1-methyl-3-ethylimidazolium chloride room temperature molten salt. J. Electrochem. Soc..

[117] Szymczak J., Legeai S., Diliberto S., Migot S., Stein N., Boulanger C., Gregory C., Draye M. (2012). Template-free electrodeposition of tellurium nanostructures in a room-temperature ionic liquid. Electrochem. Comm..

[118] Al-Salman R., Sommer H., Brezesinski T., Janek J. (2015). Template-Free electrochemical synthesis of high aspect ratio sn nanowires in ionic liquids: A general route to large-area metal and semimetal nanowire arrays?. Chem. Matter..

[119] Bartlett P.N., Cook D., (Kees) de Groot C.H., Hector A.L., Huang R., Jolleys A., Kissling G.P., Levason W., Pearce S.J., Reid G. (2013). Non-aqueous electrodeposition of p-block metals and metalloids from halometallate salts. RSC Adv..

[120] Thiebaud L., Legeai S., Ghanbaja J., Stein N. (2016). Electrodeposition of high aspect ratio single crystalline tellurium nanowires from piperidinium-based ionic liquid. Electrochim. Acta.

[121] dos Santos L.P.M., Freire R.M., Michea S., Denardin J.C., Araújo D.B., Barros E.B., Correia A.N., Lima-Neto P. (2019). Electrodeposition of 1-D tellurium nanostructure on gold surface from choline chloride-urea and choline chloride-ethylene glycol mixtures. J. Mol. Liq..

[122] Agapescu C., Cojocaru A., Cotarta A., Visan T. (2013). Electrodeposition of bismuth, tellurium, and bismuth telluride thin films from choline chloride–oxalic acid ionic liquid. J. Appl. Electrochem..

[123] Hsiu S.-I., Sun I.-W. (2004). Electrodeposition behaviour of cadmium telluride from 1-ethyl-3-methylimidazolium chloride tetrafluoroborate ionic liquid. J. Appl. Electrochem..

[124] Tsai R.W., Hsieh Y.T., Chen P.Y., Sun I.W. (2014). Voltammetric study and electrodeposition of tellurium, lead, and lead telluride in room-temperature ionic liquid 1-ethyl-3-methylimidazolium tetrafluoroborate. Electrochim. Acta.

[125] Golgovici F., Cojocaru A., Anicai L., Visan T. (2011). Surface characterization of BiSbTe thermoelectric films electrodeposited from chlorides aqueous solutions and choline chloride based ionic liquids. Mater. Chem. Phys..

[126] Mares M.L., Golgovici F., Visan T. (2013). RDE voltammetry and impedance spectroscopy studies of Sb, Te and SbTe electrodeposition from choline chloride–urea ionic liquids. Chalcogenide Lett..

[127] Catrangiu A.-S., Sin I., Prioteasa P., Cotarta A., Cojocaru A., Anicai L., Visan T. (2016). Studies of antimony telluride and copper telluride films electrodeposition from choline chloride containing ionic liquids. Thin Sol. Film..

[128] Liu J.F., Wang S.-X., Yang K.-Z. (1997). Electrodeposition and characterization of thallium(III) oxide films. Thin Sol. Film..

[129] Abd El-Halim A.M., Khalin R.M. (1984). Electrodeposition of thallium powder from sulphate baths. Surf. Tech..

[130] Tsirlina G.A., Petrii O.A., Vassiliev S.Y. (1996). Multiphase electrocrystallization in the thallium oxide system: Simultaneous nucleation on sites of different nature. J. Electroanal. Chem..

[131] Deferm C., Malaquias J.C., Onghena B., Banerjee D., Luyten J., Oosterhof H., Fransaer J., Binnemans K. (2019). Electrodeposition of indium from the ionic liquid trihexyl(tetradecyl)phosphonium chloride. Green Chem..

[132] Alcanfor A.A.C., dos Santos L.P.M., Dias D.F., Correia A.N., de Lima-Neto P. (2017). Electrodeposition of indium on copper from deep eutectic solvents based on choline chloride and ethylene glycol. Electrochim. Acta.

[133] Liu Y., Liu K., Luo L., Yuan L., Chai Z., Shi W. (2018). Direct separation of uranium from lanthanides (La, Nd, Ce, Sm) in oxide mixture in LiCl-KCl eutectic melt. Electrochim. Acta.

[134] Geran S., Chamelot P., Serp J., Gibilaro M., Massot L. (2020). Electrochemistry of uranium in molten LiCl-LiF. Electrochim. Acta.

[135] Nikitenko S.I., Cannes C., Le Naour C., Moisy P., Trubert D. (2005). Spectroscopic and electrochemical studies of U(IV)−Hexachloro complexes in hydrophobic room-temperature ionic liquids [BuMeIm][Tf2N] and [MeBu3N][Tf2N]. Inorg. Chem..

[136] Takao K., Bell T.J., Ikeda Y. (2013). Actinide chemistry in ionic liquids. Inorg. Chem..

[137] Murali Krishna G., Suneesh A.S., Venkatesan K.A., Antony M.P. (2016). Electrochemical interference of some fission products during the electrodeposition of uranium oxide from 1-butyl-3-methylimidazolium chloride ionic liquid. J. Electroanal. Chem..

[138] Lopes L., Martinot L. (2002). Electrochemistry of uranium in molten phenanthrene/TBABF4 mixtures and related organic media. J. Radioanal. Nucl. Chem..

[139] Giridhar P., Venkatesan K.A., Subramaniam S., Srinivasan T.G., Vasudeva Rao P.R. (2008). Extraction of uranium (VI) by 1.1 M tri-n-butylphosphate/ionic liquid and the feasibility of recovery by directelectrodeposition from organic phase. J. Alloys Compd..

[140] Wanigasekara E., Freiderich J.W., Sun X.G., Meisner R.A., Luo H., Delmau L.H., Daia S., Moyer B.A. (2016). Tandem dissolution of UO_3_ in amide-based acidic ionic liquid and in situ electrodeposition of UO_2_ with regeneration of the ionic liquid: A closed cycle. Dalton Trans..

[141] Jagadeeswara R.C., Venkatesan K.A., Nagarajan K., Srinivasan T.G., Vasudeva Rao P.R. (2011). Electrodeposition of metallic uranium at near ambient conditions from room temperature ionic liquid. J. Nucl. Mater..

[142] Bhujbal A.V., Rout A., Venkatesan K.A., Bhanage B.M. (2020). Electrochemical behavior and direct electrodeposition of UO_2_ nanoparticles from uranyl nitrate dissolved in an ammonium-based ionic liquid. J. Mol. Liq..

[143] Jiang Y., Ding J., Luo L., Shi P., Wang X. (2017). Electrodepositing aluminum coating on uranium from aluminum chloride-1-ethyl-3-methylimidazolium chloride ionic liquid. Surf. Coat. Tech..

[144] Jiang Y., Fang L., Luo L., Wang S., Wang X. (2018). Deposition mechanism of aluminum on uranium in AlCl_3_-1-ethyl-3-methylimidazolium chloride ionic liquid by galvanic displacement. J. Appl. Electrochem..

[145] McNamara B.K., O’Hara M.J., Clark R.A., Morrison S.S., Soderquist C.Z., Scheele R.D. (2020). Gas-Phase Molybdenum-99 Separation from Uranium Dioxide by Fluoride Volatility Using Nitrogen Trifluoride. RSC Adv..

[146] Bertch T.C. (2014). Selective Gaseous Extraction: Research, Development and Training for Isotope Production.

[147] Krohn K.A., Moerlein S.M., Link J.M., Welch M.J. (2012). Hot Atom Chemistry and Radiopharmaceuticals. AIP Conf. Proc..

[148] Czerwinski Κ.R., Kacher C.D.D., Gregorich Κ.E., Hamilton T.M.M., Hannink Ν.J., Kadkhodayan Β.A., Kreek S.A.A., Lee D.M.M., Nurmia M.J.J., Türler A. (1994). Solution Chemistry of Element 104: Part II. Liquid-Liquid Extractions with Tributylphosphate. Radiochim. Acta.

[149] Czerwinski K.R., Gregorich K.E., Hannink N.J., Kacher C.D., Kadkhodayan B.A., Kreek S.A., Lee D.M., Nurmia M.J., Türler A., Seaborg G.T. (1994). Solution Chemistry of Element 104: Part I. Liquid-Liquid Extractions with Triisooctylamine. Radiochim. Acta.

[150] Szilard L., Chalmers T.A. (1934). Chemical Separation of the Radioactive Element from Its Bombarded Isotope in the Fermi Effect. Nature.

[151] Dorhout J.M., Wilkerson M.P., Czerwinski K.R. (2019). Irradiation and Isolation of Fission Products from Uranium Metal-Organic Frameworks. J. Radioanal. Nucl. Chem..

[152] Dorhout J.M., Wilkerson M.P., Czerwinski K.R. (2019). A UO2 -Based Salt Target for Rapid Isolation of Fission Products. J. Radioanal. Nucl. Chem..

[153] Cesini G., Lucarni G., Rustichelli F. (1975). Evaluation of Fission Fragment Ranges in Any Medium. Nucl. Instruments Methods.

[154] Marks N.A., Carter D.J., Sassi M., Rohl A.L., Sickafus K.E., Uberuaga B.P., Stanek C.R. (2013). Chemical Evolution via Beta Decay: A Case Study in Strontium-90. J. Phys. Condens. Matter.

[155] Kuo E.Y., Qin M.J., Thorogood G.J., Huai P., Ren C.L., Lumpkin G.R., Middleburgh S.C. (2017). Transmutation of ABO_4_ Compounds Incorporating Technetium-99 and Caesium-137. Model. Simul. Mater. Sci. Eng..

